# Light-Driven Hexagonal-to-Cubic Phase Switching in Arylazopyrazole Lyotropic Liquid Crystals

**DOI:** 10.1021/jacs.4c02709

**Published:** 2024-04-29

**Authors:** Beatrice E. Jones, Jake L. Greenfield, Nathan Cowieson, Matthew J. Fuchter, Rachel C. Evans

**Affiliations:** † Department of Materials Science & Metallurgy, University of Cambridge, 27 Charles Babbage Road, Cambridge, CB3 0FS, U.K.; ‡ Diamond Light Source, Harwell Science and Innovation Campus, Didcot, Oxfordshire OX11 0DE, U.K.; § Department of Chemistry, Molecular Sciences Research Hub, White City Campus, Imperial College London, 82 Wood Lane, London, W12 7SL, U.K.; ∥ Institut für Organische Chemie, Universität Würzburg, Am Hubland, 97074 Würzburg, Germany

## Abstract

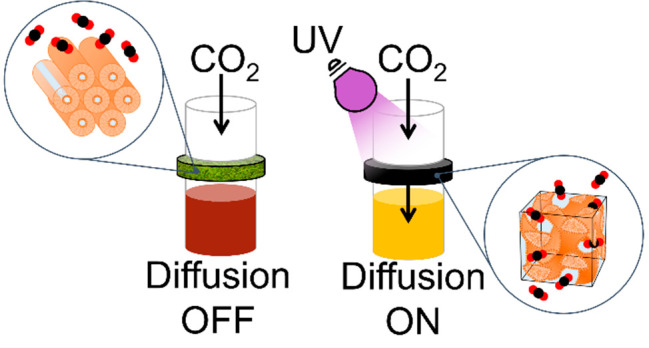

Photoinduced manipulation of the nanoscale molecular structure and organization of soft materials can drive changes in the macroscale properties. Here we demonstrate the first example of a light-induced one- to three-dimensional mesophase transition at room temperature in lyotropic liquid crystals constructed from arylazopyrazole photosurfactants in water. We exploit this characteristic to use light to selectively control the rate of gas (CO_2_) diffusion across a prototype lyotropic liquid crystal membrane. Such control of phase organization, dimensionality, and permeability unlocks the potential for stimuli-responsive analogues in technologies for controlled delivery.

Molecular self-assembly is a simple yet powerful method to construct materials with complex nanostructured architectures. Lyotropic liquid crystals (LLCs) possess long-range order from the self-assembly of amphiphilic molecules in a solvent. LLCs exhibit numerous phases that are sensitive to the molecular structure of the amphiphile and the local packing present. This makes them attractive for diverse applications, such as drug delivery,^1,2^ protein crystallization,^3^ and templates for nanostructured materials.^4^ To impart external control, light is an ideal stimulus, as it is noninvasive and can be easily controlled in time and space. Light-addressability can be achieved by introducing photoswitchable chromophores into an amphiphile to produce a photosurfactant (PS) whose molecular shape and self-assembly can be modified using light.^5−7^ Azobenzene (Azo) is the most extensively studied photoswitch for PS molecules. It exhibits *trans* (*E*) to *cis* (*Z*) isomerization under UV light, forming a photostationary state (PSS) that can be reversed by using blue light or heat. More recently, arylazopyrazoles (AAPs), where one of the phenyl rings of azobenzene is replaced by a pyrazole, have emerged as promising alternatives due to their improved performance, including quantitative photoswitching and greater thermal stability in the *Z* state.^8−10^ However, examples of integration of AAPs into surfactants are limited and mainly focus on the air–water interface,^11−14^ with no reports of self-assembly into LLC phases to date.

Previous demonstration of photoswitchable LCs has focused on Azo-containing thermotropic phases, which display temperature- rather than solvent-dependent self-assembly.^15−18^ While there are a handful of examples of light-induced phase switching, including smectic (2-D) to cubic (3-D),^19,20^ or columnar (1-D) to smectic (2-D),^21^ thermotropic LCs are limited by the high temperatures of these transitions (>100 °C). In contrast, due to their similarity to biological lipids, lyotropic LCs are highly sensitive to phase changes around room temperature. This increases their potential for applications under ambient conditions and improves compatibility with photoswitches (Azo or AAP), which undergo reverse (*Z*–*E*) isomerization at higher temperatures. There are fewer examples where AzoPSs have been used to create photoresponsive LLCs. Lamellar, hexagonal, or cubic phases have been formed where the symmetry of the phase is retained^6^ or dimensions are modified^22,23^ or destroyed^23−26^ upon irradiation with light. However, a light-induced change in dimensionality has yet to be achieved.

Here, we demonstrate the first room-temperature, light-induced dimensionality transition in an LLC, from a hexagonal (1-D) to an inverse bicontinuous gyroid cubic (3-D) phase. The LLCs are formed using a cationic arylazopyrazole photosurfactant (AAP-PS, Figure 1a) based upon cetyltrimethylammonium bromide (CTAB), whose physical properties can be tuned on isomerization.^27^ This creates a structural continuity in the phase due to the interconnected domains in the gyroid cubic phase, which are not present in the hexagonal phase. This dimensional control unlocks the potential for stimuli-responsive analogues of gyroid cubic structures in applications such as switchable ion conduction pathways for fuel cells,^28,29^ targeted drug delivery,^30^ or controllable DNA templating.^31^ As a proof-of-principle, we demonstrate tunable gas diffusion through the LLC using light, which could be used to create switchable membranes for enhanced control in microfluidic reactions, healthcare, or water treatment.^32,33^

**Figure 1:**
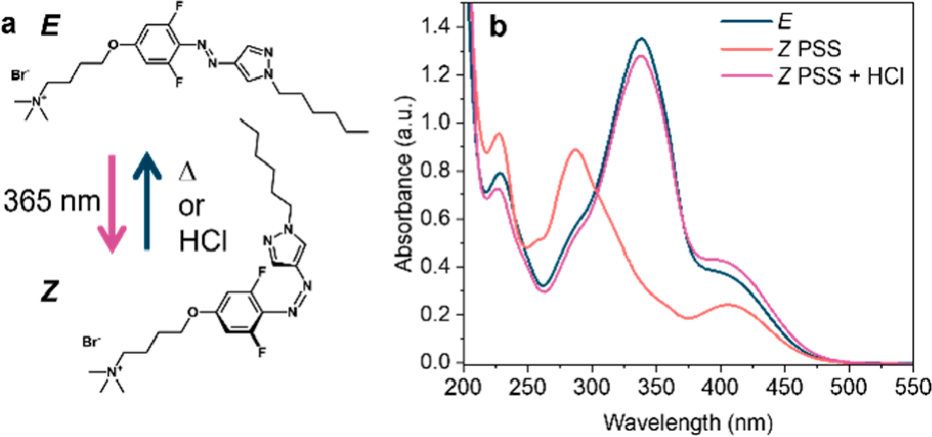
(a) Isomerization of AAP-PS from the *E* to *Z* state on UV (365 nm) light exposure results in a change in molecular shape. This can be reversed using acid or heat. (b) UV–vis absorption spectra of AAP-PS (80 μM in water) in the native, *E*, isomerized *Z*-PSS and upon reverse isomerization using HCl (after 18 h).

AAP-PS displays *E*-to-*Z* isomerization under UV (365 nm) light, reaching a PSS containing 98% *Z* isomer (Table S6), which changes the shape and polarity^27^ of the surfactant (Figure 1a). The metastable, *Z* isomer has a thermal half-life of 5.7 years at room temperature, but near-complete reverse isomerization can be catalyzed overnight with acid (HCl, excess) (Figure 1b). The critical micelle concentrations (CMCs) are 5.4 ± 2.2 and 7.8 ± 0.7 mM for the *E* and *Z* isomers, respectively (Figure S1). As expected from similar Azo-PS,^5^ the CMC in the *Z*-rich state is higher than in the *E* state due to the increased polarity on isomerization.

The effect of isomerization on micellar assemblies (20–100 mM) was investigated by model fitting to small-angle X-ray scattering (SAXS) data (SI, Section 4). AAP-PS in the *E* state forms oblate ellipsoidal micelles, which become more spherical on isomerization, consistent with previous work (Figure S5).^27^ This can be attributed to a shape-change to the bent conformation, reducing the π–π stacking possible in the *E* isomer. Additionally, the change in geometry and increase in polarity of the AAP unit when going from *E* to *Z*(34) result in its incorporation into the surfactant’s hydrophilic headgroup, with the tail group comprising only the alkyl chain, thereby increasing the overall headgroup area and interfacial curvature of the micelles.^5^ Moreover, decreased charge interactions upon isomerization were also observed (Figure S5), which is supported by a decrease in the zeta (*ζ*) potential from 65 mV to 55 mV (Table S3). This decreased effective micelle charge could be due to the smaller micelle size and aggregation number, or the change in geometry may lead to additional shielding of the cationic surfactant headgroup.

The concentration of AAP-PS, in the *E* isomer, was increased in water to form LLC phases, which were determined by using a combination of SAXS and polarized optical microscopy (POM). At 10–30 wt %, the SAXS patterns show two broad peaks indicative of an isotropic micellar phase (*I*_0_), supported by a black micrograph showing there are no anisotropic, birefringent phases present (Figure 2). At >50 wt %, sharp Bragg diffraction peaks appear (Figure 2a), indicating the formation of LLC phases. From 50 to 70 wt %, the peaks have a *q* ratio of 1:√3:2, characteristic of an inverse hexagonal (*H*_II_) mesophase, observed as a birefringent, densely-packed fan pattern by POM (Figure 2b). This is analogous to the non-light-responsive analogue, CTAB, at these concentrations.^35^ At 90 wt %, the hexagonal phase is still present, but a secondary, lamellar phase (*L*_c_) appears, shown by the peaks of ratio 1*:2* and a smoke-like texture using POM (Figure 2).^36^ On increasing the temperature, AAP-PS forms a rich phase diagram (Figure 2c; see SI, Section 6 for discussion) containing *I*_0_, *H*_II_, *L*_c_, and inverse bicontinuous gyroid cubic (*Q*_II_^G^) phases, which is promising to build a system where the phase, nanostructure, and associated properties can be tuned using light.

**Figure 2:**
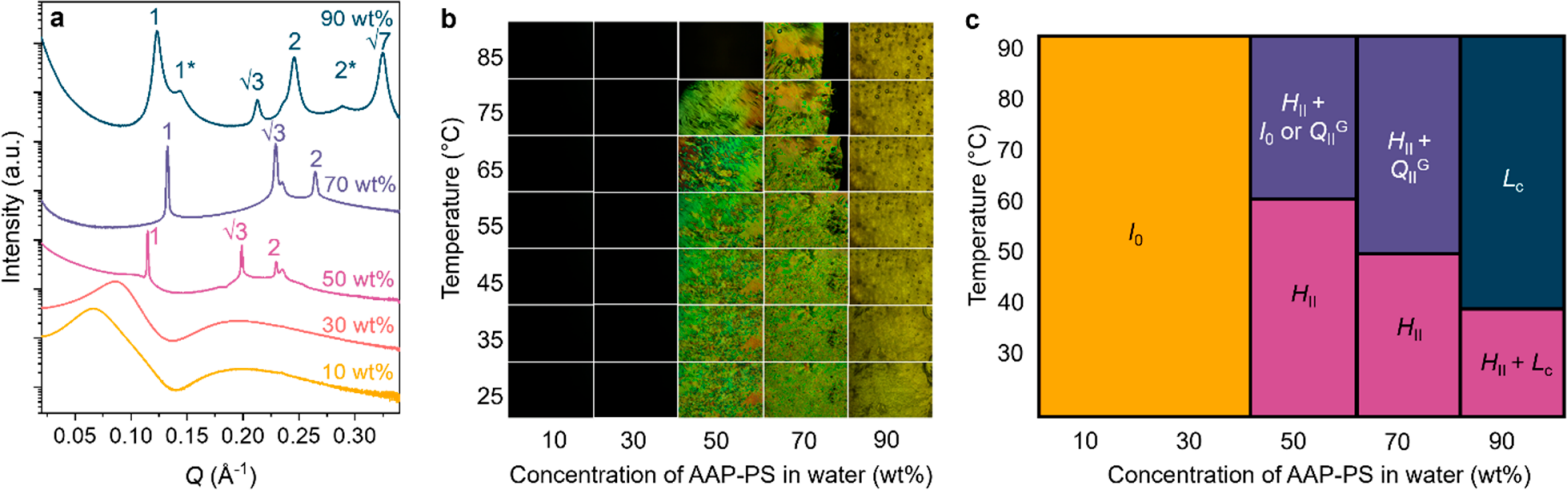
LLC formation for AAP-PS in the *E* isomer in water. (a) SAXS patterns on increasing the concentration (wt %) show the formation of Bragg peaks, characteristic of hexagonal and lamellar LLC phases. (b) POM images at increasing concentration and temperature. Black micrographs indicate isotropic phases. At >50 wt %, bright patterns support hexagonal or lamellar LLC assignments. (c) Binary phase diagram for AAP-PS showing isotropic micellar (*I*_0_), inverse hexagonal (*H*_II_), inverse bicontinuous gyroid cubic (*Q*_II_^G^), and lamellar crystalline (*L*_c_) phases.

The effect of UV irradiation on AAP-PS-water LLCs was next investigated. Before SAXS measurement, LLC phases were UV-irradiated for 3.5 h. To estimate the isomerization percentages, identical AAP-PS-D_2_O LLCs were analyzed using ^1^H NMR spectroscopy (Table S6). For isotropic micellar phases (10–30 wt % AAP-PS), irradiation resulted in >80% of the *Z* isomer and led to a shift of the SAXS interaction peaks to higher *q* values and a decrease in the overall scattering intensity (Figure S7). This suggests a decrease in the micelle size and number, as observed at lower concentrations. At 50 wt %, the hexagonal phase remains after irradiation (Figure S7), but with a significantly lower isomerization degree (54% *Z*). The degree of photoisomerization in different LLC mesophases has not yet been explored, but a decline in the achievable isomerization is expected from studies on thermotropic, Azo-containing LCs.^37^ The peaks are shifted to lower *q*, indicating an increase in the spacing between cylindrical micelles, consistent with the larger headgroup area due to inclusion of the *Z*-isomer of the AAP photoswitch. At 70 wt %, irradiation leads to loss of the hexagonal phase and formation of an isotropic micellar phase.

Excitingly, for LLCs containing 90 wt % AAP-PS, UV irradiation results in a transition from a hexagonal phase to a mixed phase containing mostly an inverse bicontinuous gyroid cubic phase, observed by the emergence of a new set of peaks with ratio √6:√8:√14:√16:√20 and a decrease in the intensity of the hexagonal peaks (Figure 3a). This phase transition corresponds to a decrease in curvature of the amphiphile bilayer, consistent with observations at lower concentrations due to the increase in effective headgroup area. This is significant, as it is associated with a dimensionality change from 1-D micellar rods in a hexagonal arrangement to a 3-D gyroid cubic structure (Figure 3b), containing interpenetrating bicontinuous networks of water and amphiphile. To the best of our knowledge, this light-driven change in long-range dimensionality has never been observed at room temperature or in an LLC. Using POM, the light-induced phase change can be controlled by light with high spatial selectivity using a mask (Figure S9). Furthermore, the changes can be reversed using acid, where the reformation of birefringent textures was observed partially after 18 h or fully after a week’s storage in the dark (Figure 3c). We have produced a system that displays a reversible and spatially controllable phase transition on irradiation with light, associated with a dimensionality change from 1- to 3-D.

**Figure 3:**
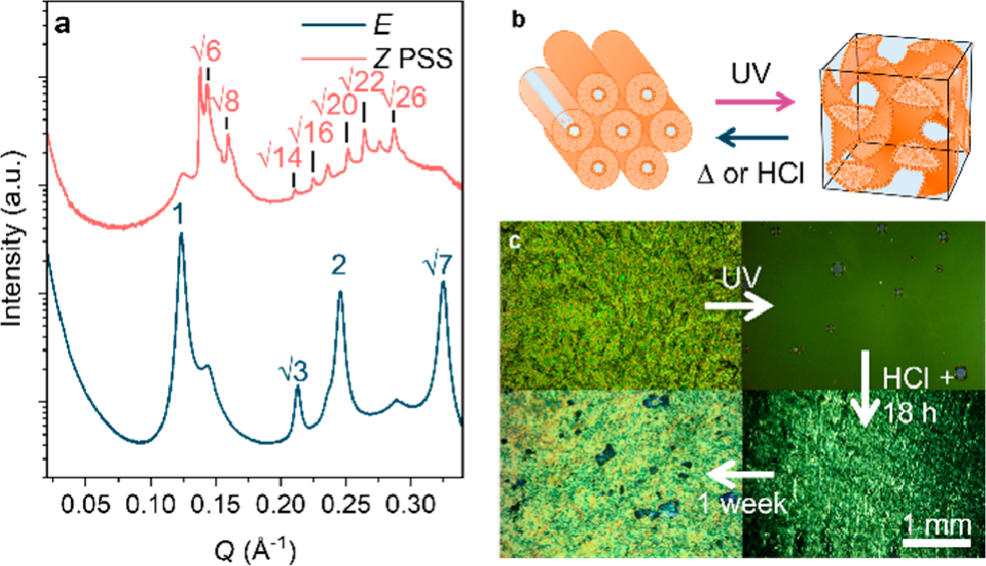
Isomerization of AAP-PS LLCs (90 wt % in water). (a) After UV irradiation, the SAXS patterns reveal a phase change from hexagonal to cubic (shown schematically in (b)). (c) POM micrographs showing birefringent, hexagonal-to-isotropic cubic transition on UV light irradiation. The birefringent phase can be reformed using acid.

The hexagonal-to-gyroid cubic phase transition is associated with structural reorganization and the formation of an interconnected bicontinuous network of water and amphiphile layers. To demonstrate the utility of this change, we designed a photoswitchable diffusion membrane to control the rate of flow of carbon dioxide (CO_2_) gas (full details in the SI, Section 1.10). CO_2_ was produced *in situ* by reacting sodium carbonate with HCl at a steady rate and fed across an LLC membrane into a bicarbonate indicator, which displays a red-to-yellow color change when the CO_2_ level increases above atmospheric concentration (0.04%). Color changes were used to monitor the diffusion rate of CO_2_ across unirradiated and UV-irradiated AAP-PS membranes. Following UV irradiation, the color changed after 5.5 min of CO_2_ addition (Figure 4a), which is much faster than the 10 min for an unirradiated membrane (Figure 4a, Supporting Video). This color change was analyzed quantitatively using UV–vis absorbance spectroscopy to follow the bicarbonate peak at 575 nm, which varies according to the solution pH to monitor the diffusion of CO_2_ into the indicator. A decrease in the peak absorbance occurs with increasing duration of CO_2_ addition, with a faster decrease for the UV-irradiated membrane than for the unirradiated membrane (Figure 4c). The rate of change of the ratio of the absorbance at 575 nm (pH-sensitive peak) to 434 nm (reference peak) over time was used to estimate the rate of diffusion of CO_2_ through the membrane. This was comparable in the UV-irradiated AAP-PS membrane and a reference membrane that contains no LLC (−0.024 ± 0.003 and −0.025 ± 0.007 min^–1^), respectively. However, the rate for the unirradiated membrane is 67% lower (−0.016 ± 0.001 min^–1^, Figure S17).

**Figure 4:**
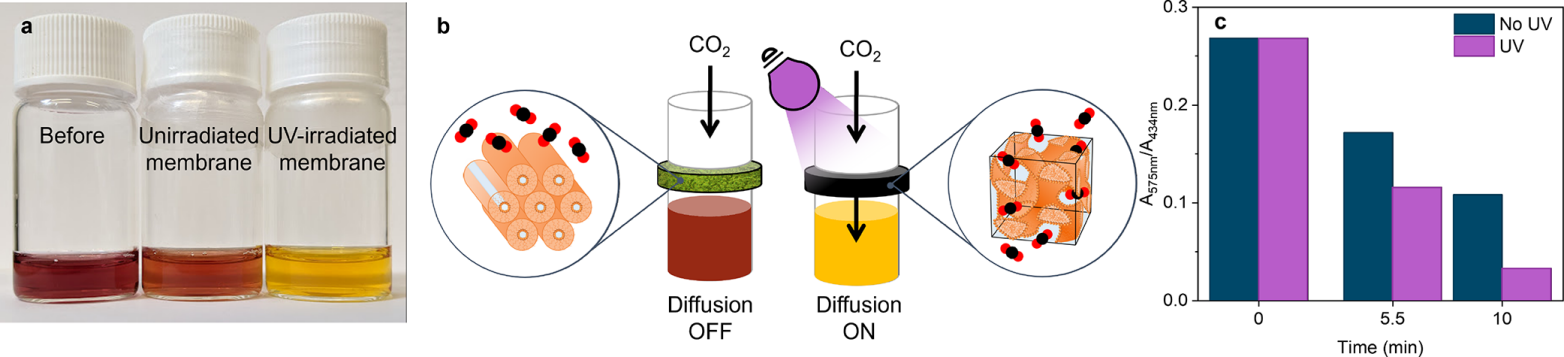
Effect of isomerization on diffusion of CO_2_ across an AAP-PS LLC membrane (90 wt %). (a) Color change of bicarbonate indicator after 10 min of CO_2_ flow across the UV-irradiated membrane (red-to-yellow) and nonirradiated membrane (red-to-orange). (b) Schematic diagram showing the effect of the hexagonal-to-bicontinuous gyroid cubic phase transition on the diffusion rate. (c) Variation of the ratio of the absorbance peaks (575 nm/434 nm) with time for CO_2_ diffusion across the membrane, for unirradiated (no UV) and UV-irradiated samples. A faster decrease in the absorption ratio indicates a greater rate of diffusion of CO_2_ across the UV-irradiated membrane.

This key result demonstrates that the hexagonal-to-inverse bicontinuous gyroid cubic LLC phase transition on UV irradiation of AAP-PS (90 wt %) leads to formation of interconnected domains that produce a structural continuity. This results in rapid diffusion of CO_2_ across the bicontinuous structure, at a rate comparable to that without the LLC present. However, in a nonirradiated sample, diffusion is limited by the intersection of cylindrical micelles between different domains of the hexagonal phase, which significantly decreases the rate of diffusion. We can therefore use light to create an open, porous network in the LLC membranes and resultantly “switch-on” diffusion. With further development, such an approach would allow for the use of controlled flow catalysis or chemistry in microfluidic systems using these as diffusion gates. However, the potential applications of these materials are not limited to membranes. Gyroid cubic structures have been used as ion conduction pathways for fuel cells,^28,29^ drug delivery,^30^ and DNA templating,^31^ and the creation of a stimulus-responsive analogue to these could unlock a new generation of functional materials for nanostructure control.

## Supporting Information Available

Materials and experimental methods; critical micelle concentrations; additional UV–vis absorption spectroscopy data; SAXS data and fits for AAP-PS micelles; zeta potential data for AAP-PS micelles; LLC phase characterization; NMR studies to determine percentage isomerized in AAP-PS LLCs; membrane diffusion studies (PDF)
Membrane diffusion studies (MP4)
