# Supplementary material for: Light-Driven Hexagonal-to-Cubic Phase Switching in Arylazopyrazole Lyotropic Liquid Crystals
Source: J Am Chem Soc. 2024 Apr 29;146(18):12315–9. doi: 10.1021/jacs.4c02709 (PMC11082889; doi:10.1021/jacs.4c02709)
Supplement: Supplementary file 1 — ja4c02709_si_001.pdf [file ja4c02709_si_001.pdf]

Supporting Information

Light-driven hexagonal-to-cubic phase switching in arylazopyrazole lyotropic liquid crystals

*Beatrice E. Jones,[a,b] Jake L. Greenfield,[c,d] Nathan Cowieson,[b] Matthew J. Fuchter[c] and Rachel C. Evans[a]**

[a] Department of Materials Science & Metallurgy, University of Cambridge, 27 Charles Babbage Road, Cambridge, CB3 0FS, United Kingdom

[b] Diamond Light Source, Harwell Science and Innovation Campus, Didcot, Oxfordshire, OX11 0DE, United Kingdom

[c] Department of Chemistry, Molecular Sciences Research Hub, White City Campus, Imperial College London, 82 Wood Lane, London, W12 7SL, United Kingdom

[d] Institut für Organische Chemie, Universität Würzburg, Am Hubland, 97074 Würzburg, Germany

* Corresponding Author: Rachel C. Evans (rce26@cam.ac.uk) and Matthew J. Fuchter (m.fuchter@imperial.ac.uk)

TABLE OF CONTENTS

1 MATERIALS AND EXPERIMENTAL METHODS 3

1.1 MATERIALS 3

1.2 SAMPLE PREPARATION 3

1.3 SAMPLE IRRADIATION 3

1.4 UV-VIS ABSORPTION SPECTROSCOPY 3

1.5 SURFACE TENSIOMETRY 4

1.6 ZETA (Z) POTENTIAL 4

1.7 SMALL-ANGLE X-RAY SCATTERING (SAXS) 4

1.8 POLARISED OPTICAL MICROSCOPY (POM) 5

1.9 ^{1}H NUCLEAR MAGNETIC RESONANCE (NMR) SPECTROSCOPY 6

1.10 MEMBRANE DIFFUSION EXPERIMENT 6

2 CRITICAL MICELLE CONCENTRATION 7

3 UV-VIS ABSORPTION SPECTROSCOPY 7

4 SAXS DATA ANALYSIS FOR AAP-PS MICELLES 10

5 Z POTENTIAL FOR AAP-PS MICELLES 14

6 LYOTROPIC LIQUID CRYSTAL PHASE CHARACTERIZATION 15

7 NMR SPECTROSCOPY TO DETERMINED PERCENTAGE ISOMERIZATION FOR IN AAP-PS-LLCS 19

8 MEMBRANE DIFFUSION STUDIES 23

9 REFERENCES 23

1 Materials and experimental methods

1.1 Materials

Sodium carbonate was provided by Acros Organics. Hydrochloric acid (37%) was supplied by Fisher Scientific. Bicarbonate Indicator Solution was supplied by Scientific Laboratory Supplied. Water was obtained from a Millipore Simak 2 water purification system. Deuterium oxide (99.9 atom% D) and dimethyl sulfoxide-d_6 (99.5 atom% D) were supplied by Sigma-Aldrich.

1.2 Sample Preparation

Photosurfactant AAP-PS was prepared according to our previously reported procedure.[1] The sample was stored in the dark and under a nitrogen atmosphere. Samples at low concentration (0.2 – 100 mM) were prepared by shaking AAP-PS with water at the appropriate concentration.

LLC phases were formed by heating the AAP-PS with either water or D_2O to 60 °C whilst stirring, until well mixed. The samples were then cooled back down to room temperature before measurement. The AAP-PS concentration was varied with respect to the solvent (10 – 90 wt%).

1.3 Sample Irradiation

To obtain the *Z*-rich PSS, samples were irradiated in a custom-built LED light box with UV (365 nm) radiation at an irradiance of 12.4 W m^{-2}. For low-concentration, micellar samples, isomerization was confirmed by diluting the samples to 83 µM and taking UV-Vis absorption spectra.

1.4 UV-Vis Absorption Spectroscopy

UV-Vis absorption spectra were taken using a Perkin Elmer Lambda 750 spectrometer with a slit width of 2 nm and a scan speed of 266.75 nm min^{-1}. Measurements were taken at 1 nm intervals from 800 – 200 nm, using quartz cuvettes with a 10 mm path length.

For Z-to-*E* isomerization measurements, 100 µL of hydrochloric acid (12 M) was added to 2 mL of AAP-PS (80 µM). Incomplete isomerization was observed immediately, with more significant change observable after 18 h storage in the dark. Note that effects from the cuvette led to a higher background in the *Z*-PSS sample, meaning a constant of 0.046 was subtracted from this trace to allow comparison with the other samples.

1.5 Surface Tensiometry

Surface tension measurements were taken using a Krüss K100 force tensiometer (Krüss GmbH, Hamburg, Germany) in a 50 mL container, filled to a depth of 6 mm. Measurements were taken using a Du Noüy ring, at a speed of 3 mm min^{-1}. Three measurements were taken for each sample and averaged, with errors given by the standard deviation. For *Z*-state measurements, the surface tensiometer was covered by a black cloth to avoid ambient light affecting the PSS. To determine the critical micelle concentration (CMC), plots of surface tension *vs.* concentration were fitted to two different straight lines, for the regions above and below the CMC. The CMC values were calculated from the intersection of these lines and error calculated using the standard error in the two straight-line regions.

1.6 Zeta (ζ) Potential

Zeta potential measurements were made using a Malvern Instruments Zetasizer. Micelle solutions of AAP-PS (20 mM) were prepared using triple-filtered water. ζ potential measurements were taken in the native, *E* state and the *Z* PSS, after irradiation for 4 hours using 365 nm light. Samples were equilibrated for 120 s at 25 °C, 3 measurements were taken with 60 s between each measurement. The 3 measurements were averaged, with the error given as the standard deviation.

1.7 Small-Angle X-ray Scattering (SAXS)

SAXS measurements were performed at the high-throughput SAXS beamline B21, Diamond Light Source (Oxfordshire, UK).[2] The X-ray beam energy was 12.4 keV and detector distance set to 4.014 m, giving a *q* range of 0.0031 – 0.34 $Å^{-1}$.

For low-viscosity micellar samples (20-100 mM), samples were loaded into a 96-well PCR plate and stored at 25°C, before injection into a quartz capillary, held at 25°C, for measurement. All the samples were moved at 1 µL/s through the beam to avoid beam damage. 21 frames of 1 s exposure time were taken and the 2D diffraction patterns were radially averaged and integrated to get 1D data. The solvent background was subtracted using the ScÅtter software.[3] *Z*-PSS samples were irradiated for 12 hours, with rotation every hour to ensure full isomerization. This was confirmed using UV-Vis absorption spectroscopy before measurement.

The data were fitted using SASFit (version 0.94.11).[4] The first 50 data points were removed and separate linear fits using OriginPro (2021b) were used to assess the aggregation

behaviour in this regime (q from 0.0045 up to 0.008 $Å^{-1}$). A linear, horizontal background was set to an appropriate value. The data were fitted to ellipsoidal core-shell structures, with a Gaussian distribution around the polar radius to incorporate polydispersity into the model. Data were fitted to the polar radius, ellipsoidal radius, shell thickness and scattering length density. For higher concentration samples, a structure factor was also required to fit to the data. To achieve this, a Hayter-Penfold MSA model was fitted to the variables: hard-sphere radius, charge and volume fraction of micelles.

For AAP-PS LLCs, samples were loaded into polyethylenimine (PEI) capillaries (low viscosity) or Kapton tape. Backgrounds of water in a PEI capillary or Kapton tape alone were subtracted using ScAtter and 20 frames of 1 s exposure time were averaged to give the final data pattern. Unless stated otherwise, samples were measured at 25 °C. For temperature ramp samples, the time needed to equilibrate the sample to the given temperature was measured using a thermocouple. The sample was then held at the temperature for the time measured before taking SAXS data.

To measure isomerized LLCs, samples were irradiated in either a polyimide capillary (for 10-30 wt% in water) or in a glass vial (50-90 wt%) for 3.5 hours. The irradiation time of 3.5 hours was chosen due to the high chromophore concentrations in the LLC phases, meaning that there is high absorbance of the UV irradiation and thus low penetration depth in the system. Furthermore, the high viscosity of the LLC phases limits diffusion of the isomerized surfactants. As such, 3.5 hours of irradiation was fixed to ensure that the surfactants formed *Z*-rich PSS. Samples in a capillary were transferred directly for SAXS measurements and samples in a vial were enclosed in Kapton tape before SAXS measurement.

For LLC phase identification, OriginPro (2021b) was used to pick the peak positions. The ratio of the q values at the peak positions relative to the first peak (q_0) were used to characterize the LLC phase present.

1.8 Polarised Optical Microscopy (POM)

POM micrographs were taken using a Leica EC4 camera fitted to an Olympus BHM microscope fitted with a polarizer and analyzer at 90° to each other. Samples were placed between two glass slides and pressed to ensure they were sufficiently thin for light transmission. Unless otherwise stated, samples were measured at room temperature. For the heating ramp, samples were heated using a Linkam PE120 Peltier heat stage controlled by a

T96 LinkPad controller and cooled using a water circulation pump. Samples were heated from 25-85 °C at a rate of 5 °C min^{-1}.

For the acid-induced reverse isomerization experiments, hydrochloric acid (12 M, 5 µL) was added to a slide containing AAP-PS LLC (90 wt%, 10 mg) that had been UV-irradiated to form the *Z*-PSS. The slide was left in the dark with periodic micrographs taken to track the self-assembly into the new LLC phase formed by the *E* isomer. Note that the addition of acid modifies the concentration of the LLC from 90 to 60 wt%, but both these concentrations are expected to form the same, H_{II} phase at room temperature in the *E* isomer.

1.9 ^{1}H Nuclear Magnetic Resonance (NMR) Spectroscopy

To determine the percentage isomerization in AAP-PS-D_2O LLCs, AAP-PS was mixed with D_2O at 60 °C in concentrations from 10-90 wt%. To mimic the irradiation conditions from the SAXS study, 10-30 wt% samples were injected (30 µL) into a PEI capillary and 50-90 wt% samples were transferred to a small vial (50 mg). Samples were irradiated at 365 nm for 3.5 hours (as above). The samples were dissolved in DMSO-d_6 (500 µL) and transferred to an NMR tube for measurement. Spectra were taken using a Bruker 400 MHz spectrometer, over 16 scans. Topspin 4.1.4 was used to calibrate to the solvent peak, pick peaks and perform peak integrations.

1.10 Membrane Diffusion Experiment

A solution of sodium carbonate (200 g L^{-1}) was added to a round-bottom flask containing hydrochloric acid (2.4 M, 50 mL) at a steady rate of 1 mL min^{-1}, using a syringe pump. Carbon dioxide formed during the reaction was fed into a Franz diffusion cell, which contained bicarbonate indicator (4 mL, 9:1 dilution) separated from the incoming gas by a hydrated, semi-permeable membrane (Spectra/Por® Biotech Dialysis Membrane, 20 kD). For diffusion studies, AAP-PS (90 wt%, ~50 mg) was loaded into a ring-shaped spacer (diameter = 20 mm, thickness = 1.5 mm) and sandwiched between two layers of semi-permeable membrane to form a thin layer. The experiment was run for a reference (with no LLC sandwiched between the semi-permeable membrane), an LLC that had been stored in the dark, and a separate sample of an LLC that had been irradiated with UV light for 3 hours.

Once the carbon dioxide addition had started, the color of the bicarbonate indicator was monitored by eye, using video and using UV-Vis absorption spectroscopy after 0, 5.5 and 10 minutes of carbon dioxide addition. For the UV-Vis absorption measurements, a 500 µL aliquot of indicator was removed from the Franz cell and replaced with an equal volume of fresh

indicator. The aliquot was diluted in 1.5 mL of water and placed in a quartz cuvette for measurement.

2 Critical Micelle Concentration

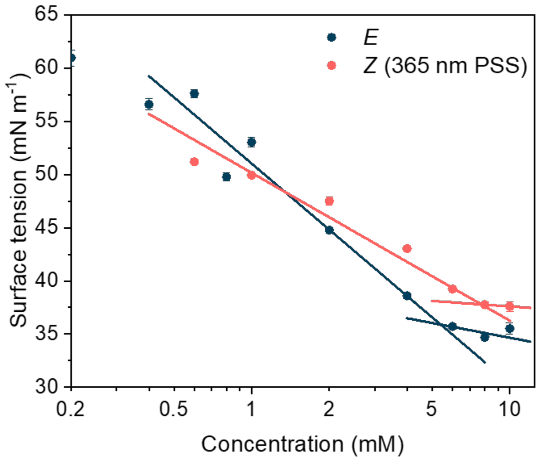

Figure S1. Determination of the CMC for AAP-PS in the *E* and *Z* isomers using surface tensiometry. The CMCs are determined by the point of intersection of the two straight-line regions. The error bars shown are the standard deviation of the mean values obtained over 3 measurements.

3 UV-Vis absorption spectroscopy

To determine the effect of micellization on the photoswitching characteristics, UV-Vis absorption spectra were taken of AAP-PS in water, at concentrations above (10 mM) and below (1 mM) the CMC. These were taken both in the native, *E* state and the Z PSS, after 45-50 minutes irradiation with UV (365 nm) light, when the UV-Vis absorption spectra no longer changed with further irradiation. Due to the high concentration, and therefore absorbance, the 10 mM samples were measured in quartz cuvette with a 0.1 mm path length, and a water background subtracted manually. 1 mM samples were measured in quartz cuvette with a 1 mm path length, with the water background subtracted by the instrument. No shifts in the absorption maxima are observed upon micellization, suggesting there is no additional H- or J-aggregation of the AAP photoswitches on aggregation into micelles. This is beneficial for retaining a high isomerization efficiency in the self-assembled state.[5]

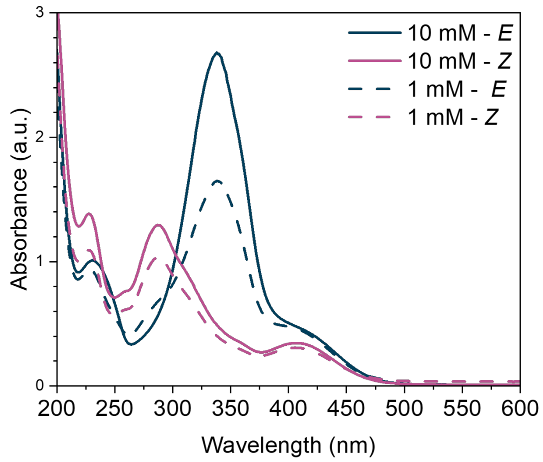

Figure S2. UV-Vis absorption spectra for AAP-PS at concentrations above (10 mM) and below (1 mM) the CMC in water. Samples were measured in the native, *E* state and the irradiated, photostationary, *Z* state.

Table S1. Variation in the peak positions of the absorption maximum (λ_{abs}^{max}) in the native *E* and *Z* PSS states for concentrations above (10 mM) and below (1 mM) the CMC, as determined using the UV-Vis absorption spectra shown in Figure S2.

Isomer	λ_{abs}^{max} Above CMC (10 mM)	λ_{abs}^{max} Below CMC (1 mM)
E	338	338
Z PSS	287	288

To determine that the AAP-PS micelle solutions had isomerized before SAXS measurement (for concentrations 20 – 100 mM) in the *Z* PSS, the UV-Vis absorption spectra were taken. The highest concentration sample (100 mM) was used, as this is expected to have the slowest isomerization, due to the high absorbance of the more concentrated sample. This was diluted to 83 µM, and 1 mL of sample was placed in a quartz cuvette. Spectra were taken from 800 – 200 nm using a slit width of 1 nm and compared to spectra for AAP-PS at the same concentrations in the native, *E* state to determine that photoisomerization had taken place.

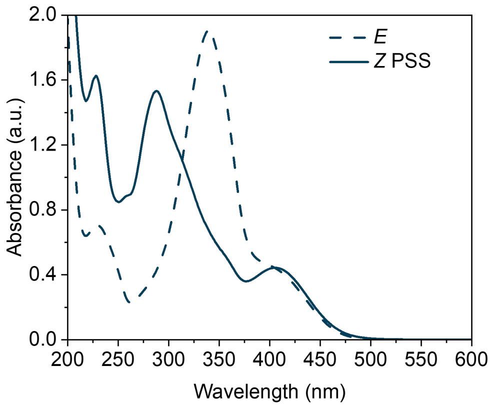

Figure S3. UV-Vis absorption spectra of AAP-PS (83 μM) after irradiation with UV light to form the *Z* PSS, before measurement using SAXS.

4 SAXS data analysis for AAP-PS micelles

The SAXS scattering profiles were modelled in SASfit software (version 0.94.11).[4] This minimizes the goodness-of-fit, X_r^2, by adjusting the model parameters, to match the model intensity with the measured intensity. Goodness-of-fit, X_r^2 is defined as:

$$X_r{}^2 = \frac{1}{(N-M)} \sum_{i=1}^{N} \left[\frac{I_{exp}(q_i) - I_{mod}(q_i)}{\sigma_{exp}(q_i)} \right]^2 \quad \text{Eq. S1}$$

where $I_{exp}(q)$ and $I_{mod}(q)$ are the measured and modelled intensities, respectively, and $\sigma_{exp}(q)$ is the uncertainty on the intensity.

The model intensity, $I_{mod}(q)$ is defined as:

$$I_{mod}(q) = \sum_{i=1}^{N} \left\{ \left[\int_a^b P(q,x) f(x)\, \mathrm{d}x \right] S(q) \right\} \quad \text{Eq. S2}$$

where $P(q, x)$ is the form factor, describing the shape of the scatterer, $f(x)$ is a shape parameter distribution, and $S(q)$ is the structure factor, describing interactions between the scatterers.

The form factor for the ellipsoid of revolution in SASfit, $I_{ECSh}(q)$ is defined by:

$$I_{ECSh}(q) = \int_0^1 [F(q, R_p, R_{eq}, t, \mu)]^2 \mathrm{d}\mu \quad \text{Eq. S3}$$

with

$$F(q, R_p, R_{eq}, t, \mu) = (\eta_{core} - \eta_{shell}) V_c \left[\frac{3 j_1(x_c)}{x_c} \right] + (\eta_{shell} - \eta_{sol}) V_t \left[\frac{3 j_1(x_t)}{x_t} \right] \quad \text{Eq. S4}$$

$$j_1(x) = \frac{\sin(x) - x\cos(x)}{x^2}$$

$$x_c = Q\sqrt{R_p^2\,\mu^2 + R_e^2(1-\mu^2)}$$

$$x_t = Q\sqrt{(R_p+t)^2\mu^2 + (R_e+t)^2(1-\mu^2)}$$

$$V_c = \frac{4}{3}\pi R_p R_e^2$$

$$V_t = \frac{4}{3}\pi(R_p + t)(R_e + t)^2$$

where η_{core} = scattering length density of core

η_{shell} = scattering length density of shell

η_{sol} = scattering length density of solvent

R_P = polar semi-axis of elliptical core

R_e = equatorial semi-axis of elliptical core

t = thickness of shell

V_c = volume of core

V_t = total volume of core along with shell

In previous studies, SAXS and SANS showed that AAP-PS self-assemble into oblate ellipsoidal micelles above the CMC, at concentrations from 10 to 40 mM.[1] Here, SAXS was used to study AAP-PS at increasing concentrations (20-100 mM), in the *E* state and the *Z* PSS, after irradiation with UV light and confirmation of switching using UV-Vis absorption spectroscopy (see above). The SAXS patterns fit well to core-shell ellipsoidal models, using SASFit, consistent with the previous literature for AAP-PS and analogous AzoTAB surfactants.[1,6] The shape and size of the micelles can be determined by analysing the form factor of the fits. For AAP-PS in the native, *E* state, oblate ellipsoids were formed with a higher equatorial radius (R_{eq}) than polar radius (R_p), consistent with the previous study.[1] On increasing the concentration above 20 mM, there is little increase in the size of the micelles, indicating that the number of micelles is instead increasing with increasing concentration. As observed previously at concentrations below 40 mM,[1] on irradiation with UV light, R_{eq} and R_p become similar, indicating that the micelles become more spherical in shape (Figure S5a). This transition can be attributed to two changes to the AAP-PS on isomerization. Firstly, a shape-change to the bent, 'T-shape' conformation, which prevents π stacking possible in the *E* isomer. Secondly, the change in geometry and polarity of the photoswitch, meaning that the AAP group is incorporated into the headgroup of the surfactant and thereby increasing the headgroup area and interfacial curvature in the micelles. With increasing concentration, there is a slight increase in both R_p and R_{eq} (Figure S5) suggesting that the whole spherical micelle size increases as a function of concentration, rather than preferential ellipsoidal growth in a

single direction, suggesting that these conclusions about the effect of curvature carry through to higher concentrations.

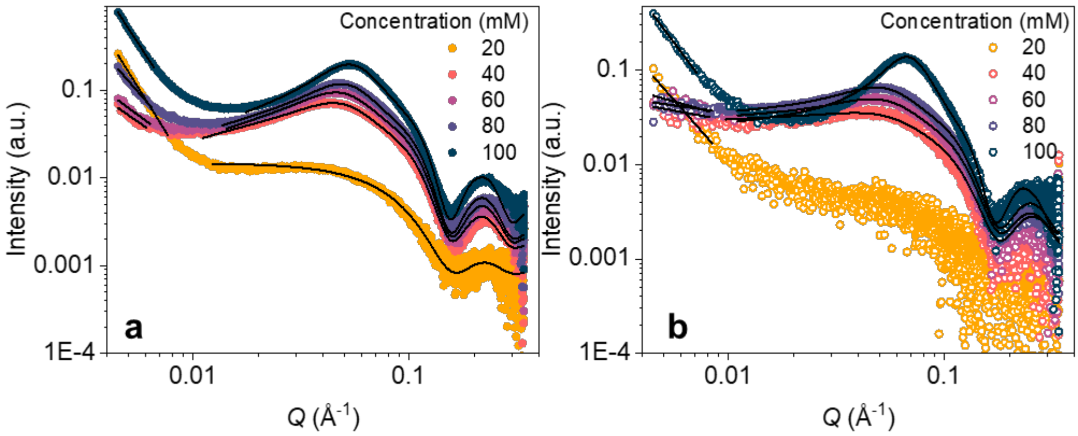

Figure S4. Changes to the shape and size of micelles of AAP-PS on increasing concentration can be determined by fitting SAXS scattering curves for AAP-PS in the (a) *E* and (b) *Z*-PSS isomers.

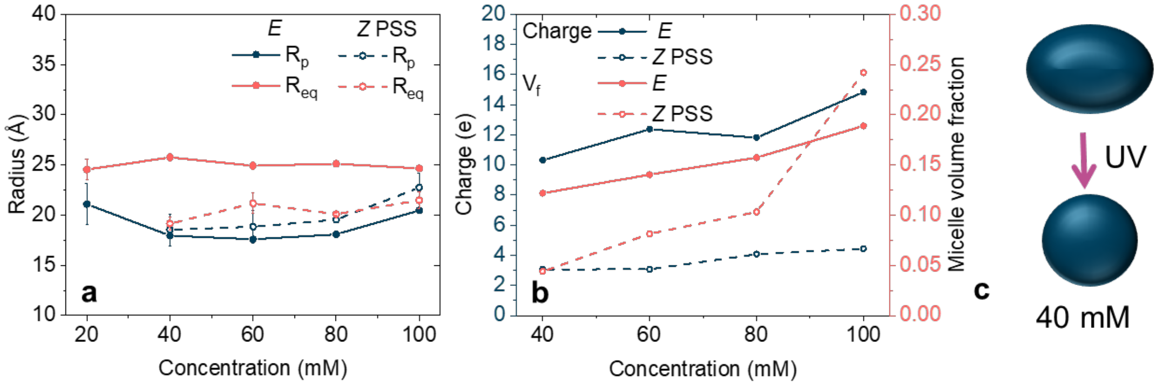

Figure S5. (a) Changes to the fitted polar (R_p) and equatorial (R_{eq}) radii of ellipsoidal micelles of AAP-PS in the *E* and *Z* isomers. (b) Changes to the fitted charge and micelle volume fraction for increasing concentration of AAP-PS in the *E* and *Z* PSS. (c) Schematic diagram showing the micelle shape on isomerization at 40 mM.

For micelles in both isomeric states, on increasing the concentration above 20 mM, interactions between the charged micelles can be seen on the SAXS curves as the formation of a hump in the scattering pattern for AAP-PS in the *E* state, at $q = 0.0045 - 0.005$ Å^{-1} (Figure S4). A Hayter-Penfold structure factor was needed to model the SAXS these interactions,[7] where the strength of the ionic interactions between the micelles is parametrized by the micelle charge given by the fitted model. From the fits, there is an increase in the charge and volume fraction of the micelles on increasing concentration, as would be expected from having a greater number of micelles interacting in solution (Figure S5). On isomerization to the *Z* PSS, the fitted charge, and therefore interactions between the micelles decreases (Figure S5). An

interaction hump only appears at concentration of 100 mM, at a higher q value in comparison to the *E* sample (0.067 $Å^{-1}$ *cf.* 0.005 $Å^{-1}$) (Figure S4). This provides further evidence that the *Z* PSS micelles are smaller than the *E* micelles, and therefore the interactions only occur at closer distances. There is also evidence of micelle destruction on isomerization, where the scattering intensity is too low to fit at 20 mM, and has no plateau from micelle scattering. This is clear in the fitted micelle volume fraction across the concentration range (Figure S5). This destruction, along with the lower charge results in fewer interactions between the micelles on isomerization.

The interactions between the micelles were further investigated by measuring the ζ potential at 20 mM in the *E* and *Z* PSS states. The lowering of the effective charge of the micelles (Table S3) suggests that isomerization of the AAP-PS, and formation of the 'T-shape' conformer, results in a shielding of the cationic surfactant head group, perhaps due to cation-π interactions that were not possible in the *E* conformer. The increase in the curvature and decrease in effective charge of AAP-PS micelles on isomerization will have an important effect on self-assembly into LLCs at higher concentrations.

The straight-line regions of the SAXS curves at low q can be used to investigate the formation of larger-scale aggregates in the system. For AAP-PS at 20 mM, a slope of q^{-4} is indicative of scattering from smooth fractal interfaces (Figure S4a and Table S2).[8] From 40 – 80 mM, this decreases to roughly q^{-2}, which is more characteristic of random-walk interactions in worm-like micelles.[9,10] At 100 mM, the scattering again is more indicative of fractal structures. This indicates that, despite the relative consistency in micelle size across the composition range, the micelle solutions are not homogeneous. There are higher-order aggregates which grow to worm-like micelle structures. On isomerization, the smooth fractal scattering for 20 and 100 mM evolves to closer to q^{-3}, indicating an increase in the roughness of the fractal aggregates, possibly due to the formation of a mixed, photostationary state. At intermediate concentrations a zero-gradient plateau forms indicating that there are only the globular, micelles contributing to the scattering. This shows that, between concentrations of 20 and 100 mM, both micelles and larger-scale structures that form are dependent upon the isomerization state of the surfactant.

Table S2. Variation in q^{-n} obtained from straight-line fits to the SAXS curves in the low-q region (shown in Figure S4) for AAP-PS of increasing concentration in the *E* and *Z* PSS states.

Concentration (mM)	*n*	
	E	*Z* PSS
20	-3.95 ± 0.04	-2.60 ± 0.14
40	-1.72 ± 0.17	-0.42 ± 0.18
60	-1.81 ± 0.13	-0.65 ± 0.15
80	-2.82 ± 0.09	-0.32 ± 0.09
100	-3.77 ± 0.04	-3.06 ± 0.06

5 ζ potential for AAP-PS micelles

Table S3. Change in the zeta potential (ζ), electrophoretic mobility and conductivity for a micellar solution of AAP-PS (20 mM) in the *E* and *Z* PSS states.

**Isomer**	**ζ (mV)**	**Mobility (µm cm/Vs)**	**Conductivity (mS/cm)**
E	65 ± 4	5.09 ± 0.32	0.368 ± 0.002
Z PSS	55 ± 2	4.31 ± 0.13	0.369 ± 0.001

6 Lyotropic liquid crystal phase characterization

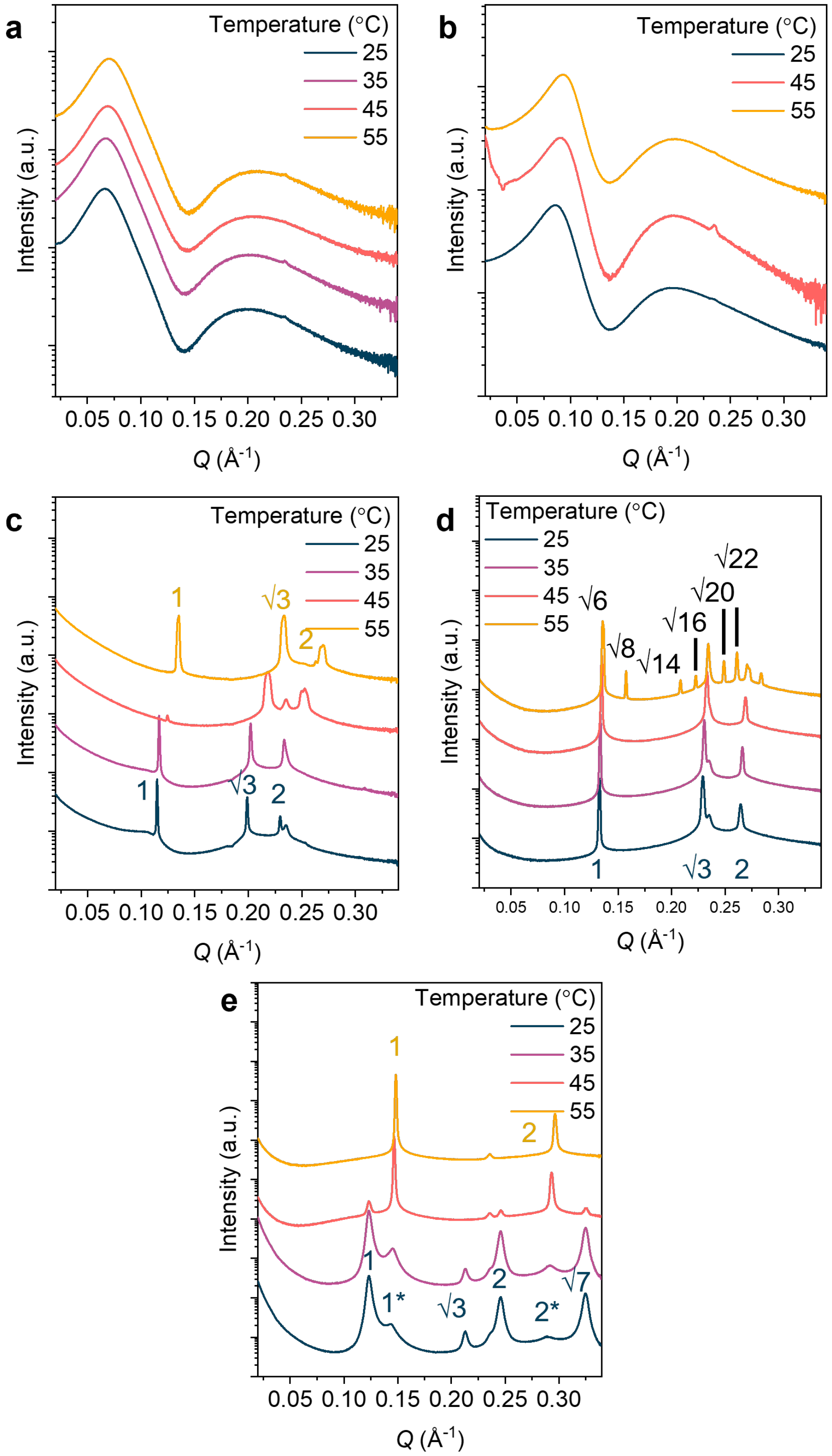

Figure S6. SAXS patterns AAP-PS as a function of temperature and concentration in water: (a) 10 wt%, (b) 30 wt%, (c) 50 wt%, (d) 70 wt% and (e) 90 wt%. The patterns have been offset for clarity.

The temperature-dependent LLC phase behavior was studied using a combination of SAXS and POM. For the isotropic micellar phases (10 – 30 wt%), increasing the temperature to 55 °C resulted in no further phase changes (Figure S6). At 50 wt%, the Bragg peaks characteristic of the hexagonal phase are retained until 55 °C. However, the POM micrographs show the emergence of an isotropic phase in equilibrium with the hexagonal phase above 65 °C, indicative of either an isotropic melt or cubic LLC phase (Figure 2b). At 70 wt%, the SAXS data show the formation of a gyroid cubic (Q_{II}^{G}) phase at 55 °C, characterized by peaks in the ratio $\sqrt{6}:\sqrt{8}:\sqrt{14}:\sqrt{16}:\sqrt{20}:\sqrt{22}$, accompanied by observation of black sections in the POM images. CTAB also forms a bicontinuous gyroid cubic phase at higher temperatures (47 °C) and concentrations (75 wt%).[11] At 90 wt%, increasing the temperature favors the lamellar phase over the hexagonal phase, observed as the dominance of the 1:2 peak ratio in the SAXS pattern and the POM showing only a smokey texture. This is due to dehydration of the head groups at high concentrations, favoring a lower-curvature lamellar phase. AAP-PS therefore forms a rich phase diagram (Figure 2c) in a manner that is comparable to CTAB.

It is worth noting that not all the peaks present in the SAXS patterns are accounted for in the assignment of the LLC phases present. In particular, the small peak at ~0.23 Å^{-1} is present in all LLC for AAP-PS in the E isomer at concentrations at 50 wt% or above. This can be attributed to an ordered crystallite, that perhaps forms at the sample surface due to dehydration effects. This argument is supported by the emergence of the same peak at higher temperature for lower concentrations samples (Figure S6b at 45 °C), as well as at high concentrations (Figure S6e at 55 °C).

Table S4. Variation in lattice parameter for AAP-PS LLCs of increasing concentration in water (wt%) at different temperatures where the AAP-PS is in the *E* isomer. Note that the phases are isotropic micellar (I_0), inverse hexagonal (H_{II}) and crystalline lamellar (L_c).

Concentration (wt%)	Phase	Lattice Parameter (Å)			
		25 °C	35 °C	45 °C	55 °C
10	I_0	N/A			
30	I_0	N/A			
50	H_{II}	63	62	58	54
70	H_{II}	55	54	54	54
90	H_{II} / L_c	59 / 44	59 / 44	59 / 42	N/A / 42

.

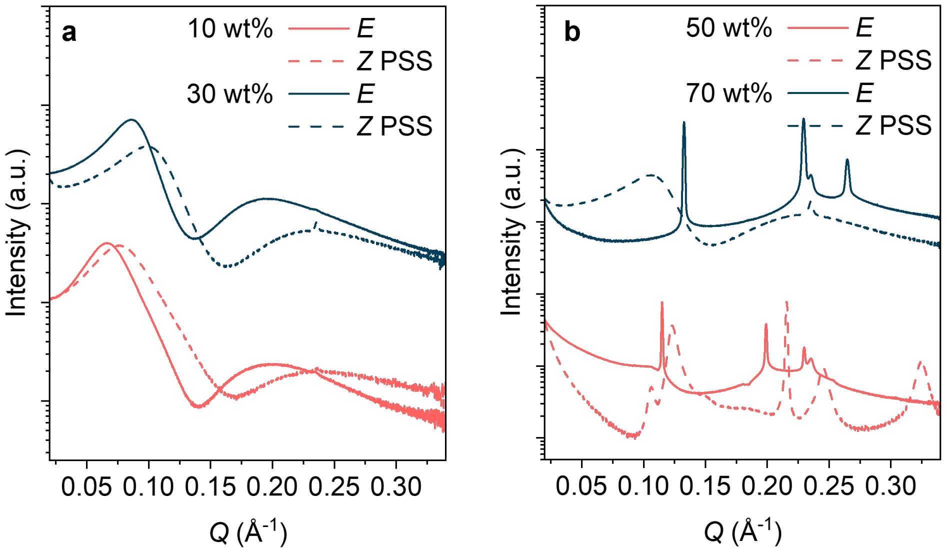

Figure S7. SAXS patterns at increasing concentrations of AAP-PS-LLCs in the *E* and *Z*-rich PSS isomeric states. (a) Concentrations of 10 – 30 wt% show a characteristic hump from isotropic micelle phases. (b) Concentration of 50 wt% shows retention of characteristic Bragg diffraction peaks from a hexagonal LLC phase, but the shift to higher *q* values on isomerization indicates an increase in the lattice parameter. 70 wt% sample shows transition from hexagonal to isotropic phase on isomerization.

Table S5. Variation in lattice parameter for AAP-PS LLCs of increasing concentration in water (wt%) where the AAP-PS is in the *Z*-rich PSS. Note that the phases are isotropic micellar (I_0), inverse hexagonal (H_{II}) and inverse bicontinuous gyroid cubic (Q_{II}^G).

Concentration (wt%)	Phase	Lattice Parameter (Å)
10	I_0	N/A
30	I_0	N/A
50	H_{II}	59
70	I_0	N/A
90	H_{II} / Q_{II}^G	58 / 53

As with the LLCs formed from AAP-PS in the *E* isomer, not all the Bragg peaks in the *Z*-rich PSS can be attributed to the assigned LLC phases. These peaks include the peak at 0.23 Å^{-1}, which was previously explained by surface dehydration effects. Furthermore, the UV-induced hexagonal to inverse bicontinuous gyroid cubic phase transition (at concentrations of 90 wt% AAP-PS) is not complete, due to the retention of some of the peaks in a ratio of 1:√3:2, characteristic of the hexagonal phase (main manuscript, Figure 3a). However, the hexagonal peaks significantly decrease in nature and are dominated by the peaks assigned to the cubic phase.

When investigated using POM, samples at all concentrations went dark after irradiation (Figure S8). This may be due to a difference in the sample loading, with the POM samples (sandwiched between two glass slides) being much thinner and more prone to surface pinning than the samples measured by SAXS, which were irradiated in the bulk and then transferred to the sample environment for measurement. It may be that the thinner path length in POM means that there is a greater degree of isomerization, which means that more of the structure is disrupted than when measured in the bulk. Alternatively, the constraint of the slides could hinder self-assembly to the new, *Z*-rich PSS LLC phase after UV irradiation.

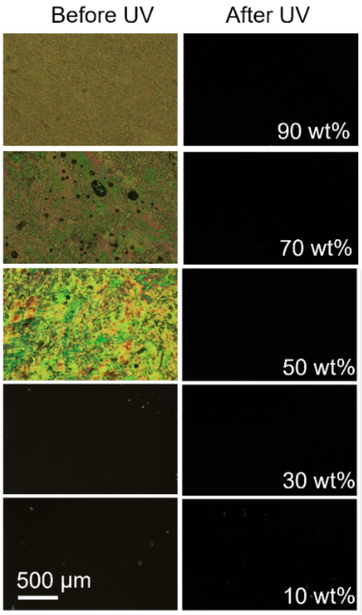

Figure S8. Changes to the POM micrographs on irradiation with UV for AAP-PS of increasing concentration in water.

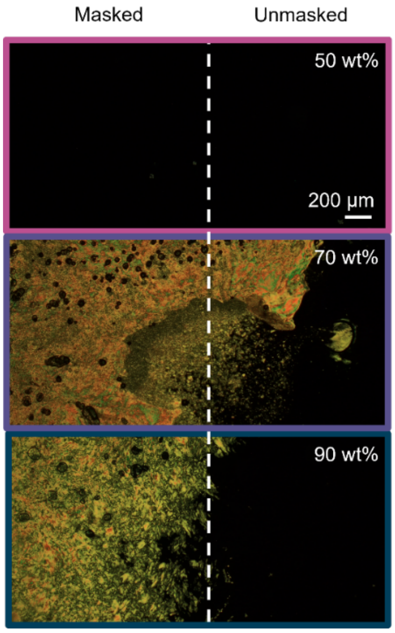

Figure S9. POM micrographs for AAP-PS-LLCs of increasing concentration (50 – 90 wt% in water) after irradiation with UV light, where half the sample has been covered with a mask.

7 NMR spectroscopy to determined percentage isomerization for in AAP-PS-LLCs

^{1}H NMR spectroscopy was used to determine the percentage isomerization for AAP-PS-LLCs into the *Z* isomer after the 3.5 hours of irradiation used for SAXS measurements. Characteristic peaks for the *E* and *Z*-rich PSS were determined using AAP-PS (10 mM) in DMSO-d_6, in the native state and the *Z*-rich PSS, after 3 hours of irradiation at 365 nm. Characteristic singlet peaks in the *E* state were present at 8.53 and 7.93 ppm. Characteristic singlet peaks of the *Z* state were present at 8.42 and 6.8 ppm. There is an additional doublet present in both the *E* and *Z* states; however, an overlap between these meant that these were not used to determine the percentage isomerized. The percentage isomerized was calculated by taking the ratio of the averaged integrals of the *Z* singlets to the sum of the averaged integrals of the *E* and *Z* singlets. The error was calculated by summing the standard deviations of the *E* and *Z* integrals.

Table S6. Percentage isomerization after 3.5 hours of irradiation under 365 nm light for AAP-PS in D_2O at increasing concentrations.

Sample	Percentage isomerization
10 mM - *E*	0 %
10 mM – *Z*	98 ± 2 %
10 wt%	97 ± 1 %
30 wt%	81 ± 4 %
50 wt%	54 ± 5 %
70 wt%	60 ± 2 %
90 wt%	71 ± 4 %

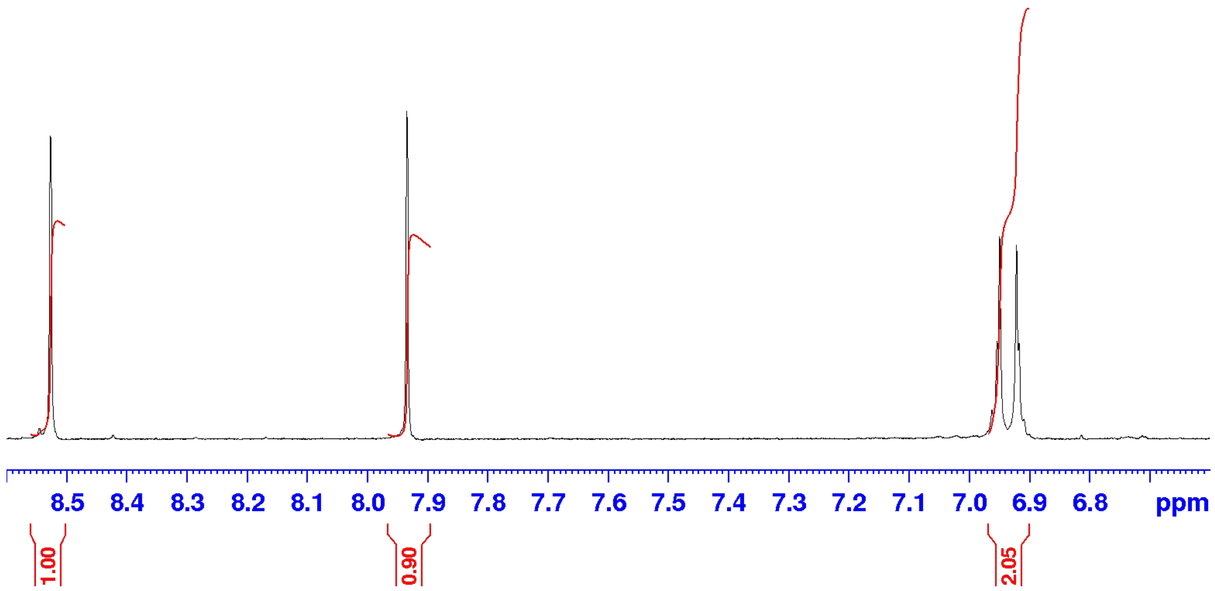

Figure S10. ^{1}H NMR spectrum for AAP-PS (10 mM) in DMSO-d6 in the *E* state.

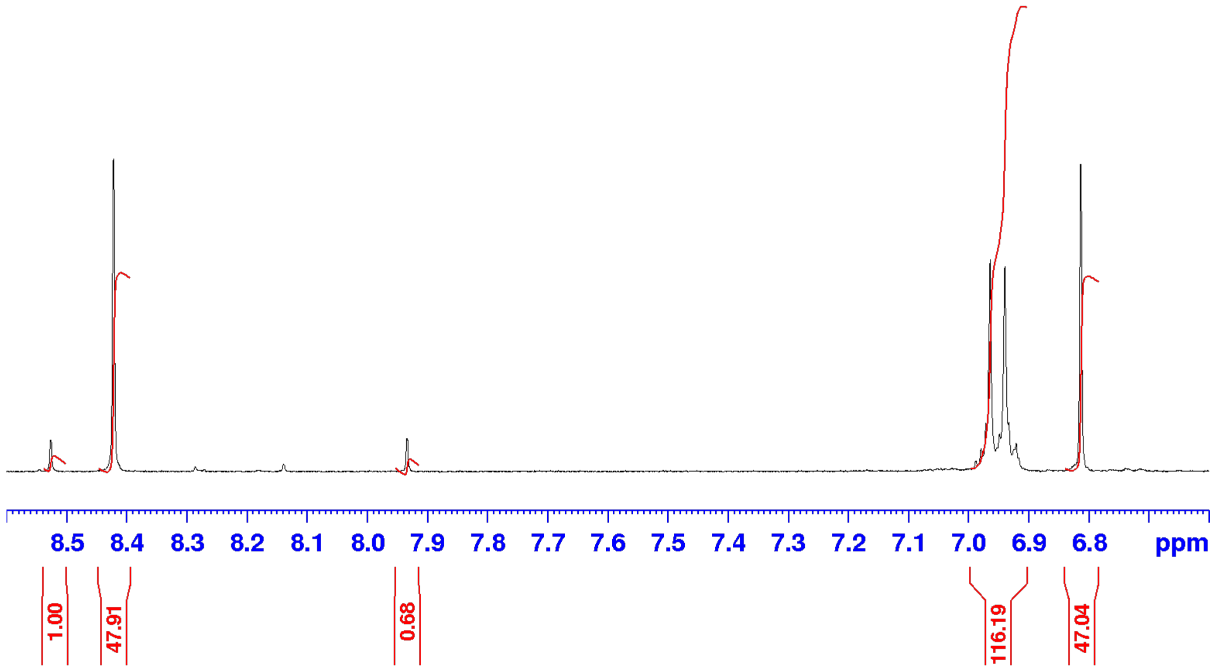

Figure S11. ^{1}H NMR spectrum for AAP-PS (10 mM) in DMSO-d6 in the Z-PSS.

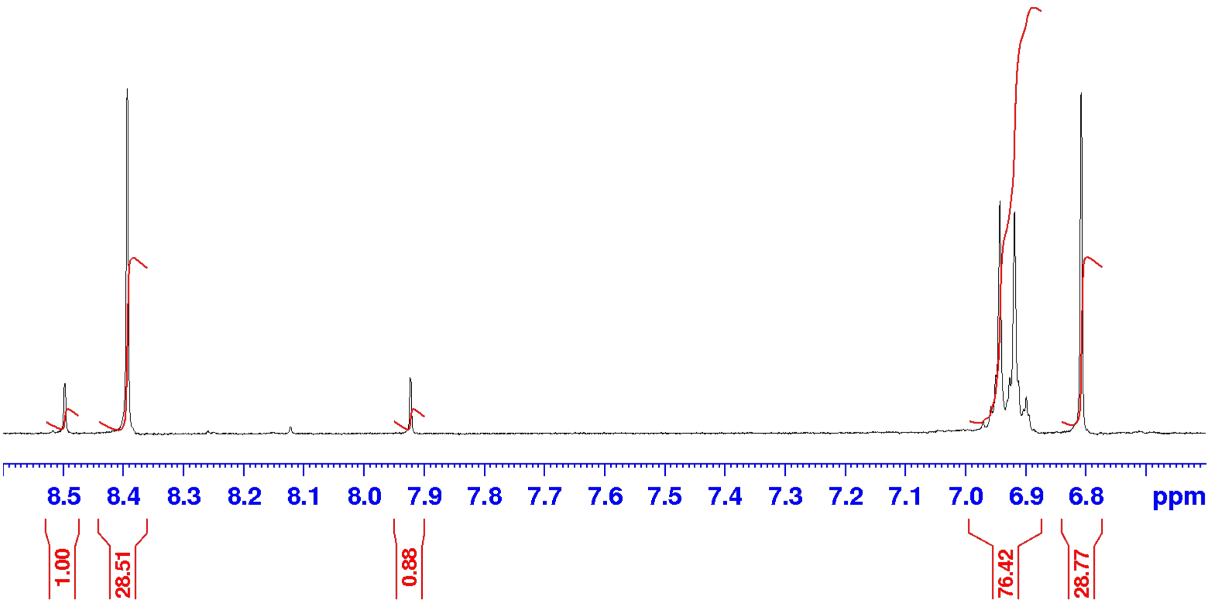

Figure S12. ^{1}H NMR spectrum for AAP-PS (10 wt%)-D_2O after 3.5 hours of irradiation, solvated in DMSO-d6.

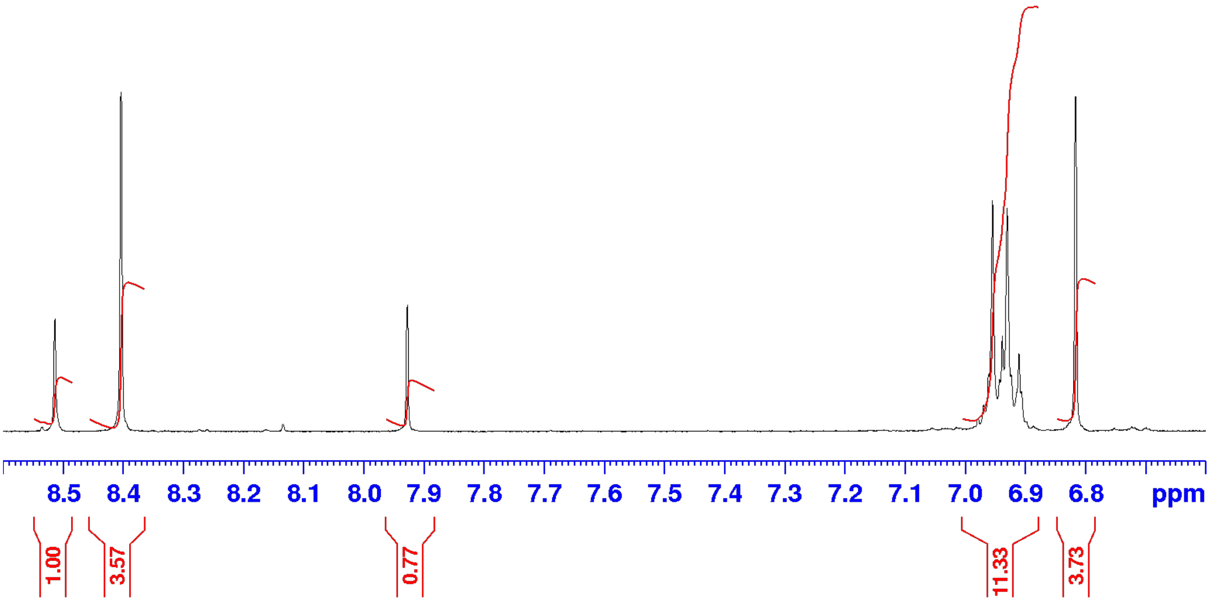

Figure S13. ^{1}H NMR spectrum for AAP-PS (30 wt%)-D_2O after 3.5 hours of irradiation, solvated in DMSO-d6.

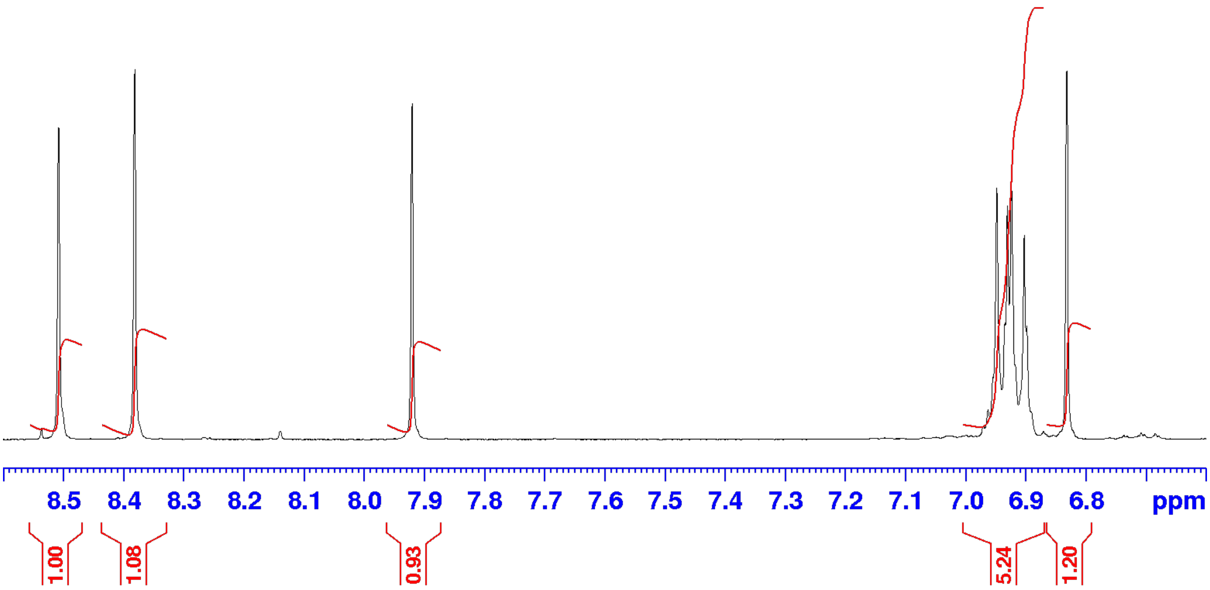

Figure S14. ^{1}H NMR spectrum for AAP-PS (50 wt%)-D_2O after 3.5 hours of irradiation, solvated in DMSO-d6.

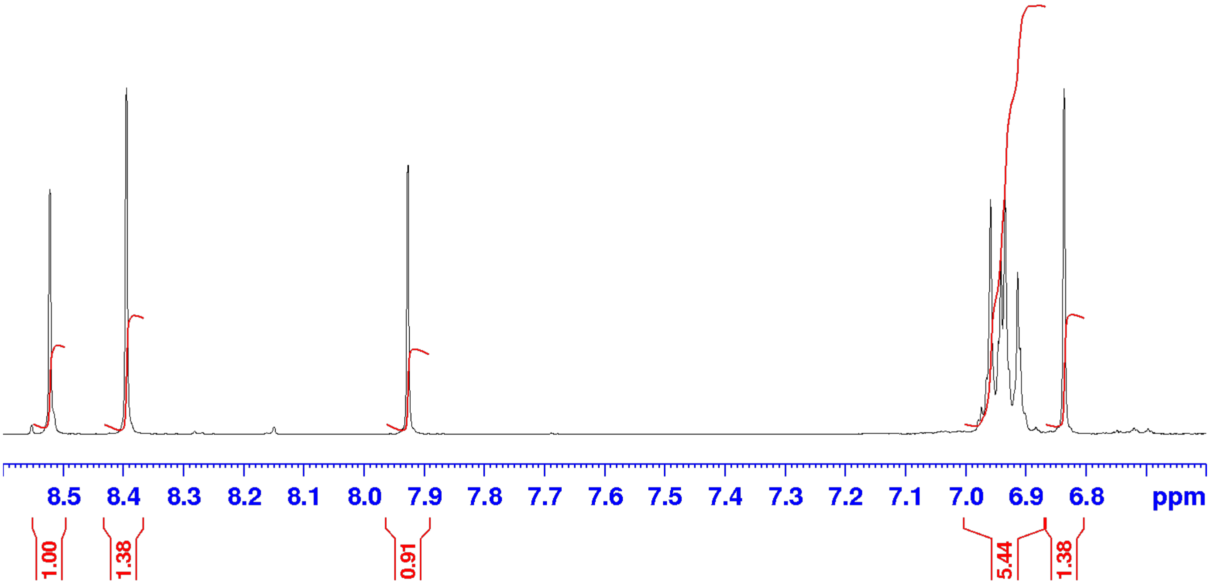

Figure S15. NMR spectrum for AAP-PS (70 wt%)-D_2O after 3.5 hours of irradiation, solvated in DMSO-d6.

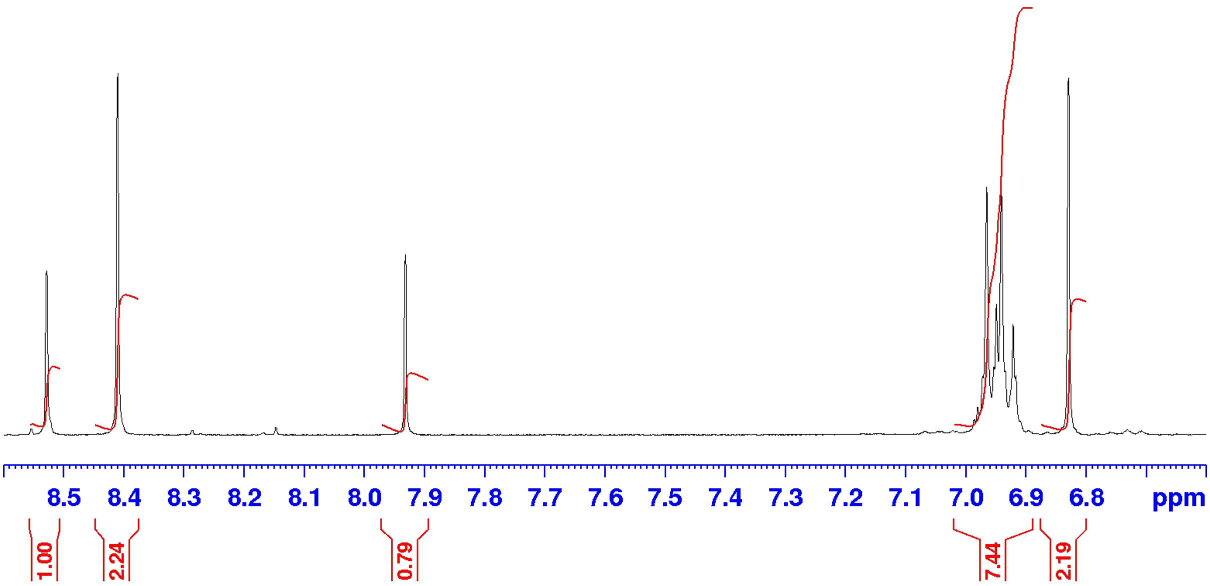

Figure S16. ^{1}H NMR spectrum for AAP-PS (90 wt%)-D_2O after 3.5 hours of irradiation, solvated in DMSO-d6.

8 Membrane diffusion studies

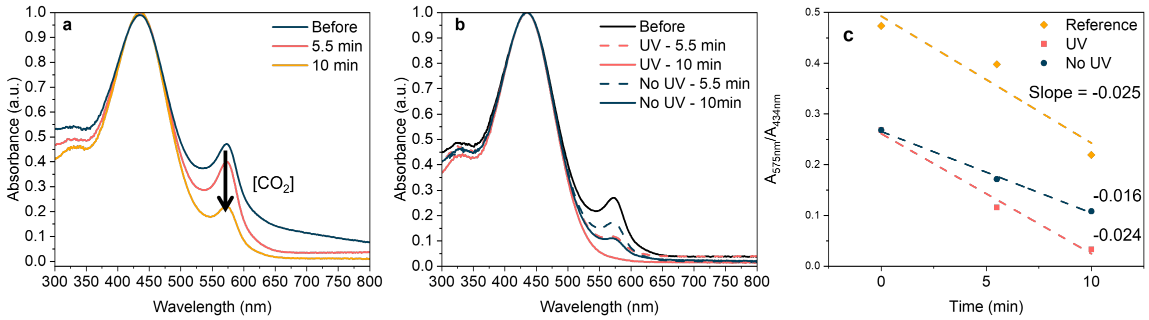

Figure S17. UV-Vis absorption spectra for bicarbonate indicator under flow of CO_2 through: (a) a reference membrane containing no AAP-PS LLC and (b) a membrane of AAP-PS LLC (90 wt%) that is unirradiated (no UV) and irradiated (UV), before CO_2, after 5.5 and 10 minutes of CO_2 flow. (c) Plot of the ratio of the absorbance at 575 nm (pH sensitive peak) to 434 nm (reference peak) as a function of time. The slope of which was used to roughly quantify the rate of CO_2 flow through the membrane.

9 References

(1) Tyagi, G.; Greenfield, J. L.; Jones, B. E.; Sharratt, W. N.; Khan, K.; Seddon, D.; Malone, L. A.; Cowieson, N.; Evans, R. C.; Fuchter, M. J.; Cabral, J. T. Light Responsiveness and Assembly of Arylazopyrazole-Based Surfactants in Neat and Mixed CTAB Micelles. *JACS Au* **2022**, *2* (12), 2670–2677. https://doi.org/10.1021/jacsau.2c00453.

(2) Cowieson, N. P.; Edwards-Gayle, C. J. C.; Inoue, K.; Khunti, N. S.; Doutch, J.; Williams, E.; Daniels, S.; Preece, G.; Krumpa, N. A.; Sutter, J. P.; Tully, A. D.; Terrill, N. J.;

Rambo, R. P. Beamline B21: High-Throughput Small-Angle X-Ray Scattering at Diamond Light Source. *J Synchrotron Radiat* **2020**, *27*, 1438–1446. https://doi.org/10.1107/S1600577520009960.

(3) Rambo, R. P. *ScÅtter*. http://www.bioisis.net/tutorial/9.

(4) Breßler, I.; Kohlbrecher, J.; Thünemann, A. F. SASfit: A Tool for Small-Angle Scattering Data Analysis Using a Library of Analytical Expressions. *J Appl Crystallogr* **2015**, *48*, 1587–1598. https://doi.org/10.1107/S1600576715016544.

(5) Giles, L. W.; Faul, C. F. J.; Tabor, R. F. Azobenzene Isomerization in Condensed Matter: Lessons for the Design of Efficient Light-Responsive Soft-Matter Systems. *Mater. Adv.* **2021,** *2*, 4152–4164. https://doi.org/10.1039/d1ma00340b.

(6) Blayo, C.; Houston, J. E.; King, S. M.; Evans, R. C. Unlocking Structure-Self-Assembly Relationships in Cationic Azobenzene Photosurfactants. *Langmuir* **2018**, *34* (34), 10123–10134. https://doi.org/10.1021/acs.langmuir.8b02109.

(7) Hayter, J. B.; Penfold, J. An Analytic Structure Factor for Macroion Solutions. *Mol Phys* **1981**, *42* (1), 109–118. https://doi.org/10.1080/00268978100100091.

(8) Teixeira, J. Small-Angle Scattering by Fractal Systems. *J Appl Crystallogr* **1988**, *21* (6), 781–785. https://doi.org/10.1107/S0021889888000263.

(9) Dreiss, C. A. Wormlike Micelles: Where Do We Stand? Recent Developments, Linear Rheology and Scattering Techniques. *Soft Matter* **2007**, *3* (8), 956–970. https://doi.org/10.1039/b705775j.

(10) Pedersen, J. S.; Schurtenberger, P. *Scattering Functions of Semiflexible Polymers with and without Excluded Volume Effects*; 1996. https://pubs.acs.org/sharingguidelines.

(11) Yamamoto, T.; Yagi, Y.; Hatakeyama, T.; Wakabayashi, T.; Kamiyama, T.; Suzuki, H. Metastable and Stable Phase Diagrams and Thermodynamic Properties of the Cetyltrimethylammonium Bromide (CTAB)/Water Binary System. *Colloids Surf A Physicochem Eng Asp* **2021**, *625,* 126859. https://doi.org/10.1016/j.colsurfa.2021.126859.
